# Targeted system approach to ethylene biosynthesis and signaling of a heat tolerant tomato cultivar; the impact of growing season on fruit ripening

**DOI:** 10.3389/fpls.2023.1195020

**Published:** 2023-06-30

**Authors:** Thao Minh Viet Nguyen, Maarten L. A. T. M. Hertog, Bram Van de Poel, Dinh Thi Tran, Bart Nicolaï

**Affiliations:** ^1^ KU Leuven, BIOSYST-MeBioS Postharvest Lab, Leuven, Belgium; ^2^ Vietnam National University of Agriculture, Faculty of Food Science and Technology, Hanoi, Vietnam; ^3^ KU Leuven Plant Institute (LPI), Leuven, Belgium; ^4^ KU Leuven, BIOSYST- Crop Biotechnics, Molecular Plant Hormone Physiology Lab, Leuven, Belgium; ^5^ Flanders Centre of Postharvest Technology, Leuven, Belgium

**Keywords:** ethylene biosynthesis, ethylene signaling, heat tolerance, ripening, postharvest storage, tomato, targeted proteomics

## Abstract

Growing tomato in hot weather conditions is challenging for fruit production and yield. Tomato cv. Savior is a heat-tolerant cultivar which can be grown during both the Vietnamese winter (mild condition) and summer (hot condition) season. Understanding the mechanisms of ethylene biosynthesis and signaling are important for agriculture, as manipulation of these pathways can lead to improvements in crop yield, stress tolerance, and fruit ripening. The objective of this study was to investigate an overview of ethylene biosynthesis and signaling from target genes to proteins and metabolites and the impact of growing season on a heat tolerant tomato cultivar throughout fruit ripening and postharvest storage. This work also showed the feasibility of absolute protein quantification of ethylene biosynthesis enzymes. Summer fruit showed the delayed peak of ethylene production until the red ripe stage. The difference in postharvest ethylene production between winter and summer fruit appears to be regulated by the difference in accumulation of 1-aminocyclopropane-1-carboxylic acid (ACC) which depends on the putative up-regulation of SAM levels. The lack of differences in protein concentrations between winter and summer fruit indicate that heat stress did not alter the ethylene biosynthesis-related protein abundance in heat tolerant cultivar. The analysis results of enzymatic activity and proteomics showed that in both winter and summer fruit, the majority of ACO activity could be mainly contributed to the abundance of ACO5 and ACO6 isoforms, rather than ACO1. Likewise, ethylene signal transduction was largely controlled by the abundance of ethylene receptors ETR1, ETR3, ETR6, and ETR7 together with the constitute triple response regulator CTR1 for both winter and summer grown tomatoes. Altogether our results indicate that in the heat tolerant tomato cv. Savior, growing season mainly affects the ethylene biosynthesis pathway and leaves the signaling pathway relatively unaffected.

## 1 Introduction

High temperature severely affects the growth and development of many climacteric fruits and in relation to that fruit quality. This makes it challenging for tropical countries to grow tomato fruit during the hot season. High temperature impairs the photosynthesis process and fruit set of heat sensitive tomato cultivars (Camejo et al., 2005; Sasaki et al, 2005). The optimum temperature for tomato ripening ranges between 20°C to 25°C (Cantwell, 2010). Above 30°C heat sensitive tomato fruit fail to ripen, resulting in abnormal color development, less softening and a decrease in ethylene evolution (Yakir et al., 1984; Brandt et al., 2006).

Ethylene is a natural plant hormone regulating many processes including fruit ripening. Ethylene biosynthesis was thoroughly elucidated by Yang and Hoffman (1984), stating from S-(5’-adenosyl)-L-methionine (SAM) 1-aminocyclopropane-1-carboxylic acid (ACC), catalyzed by ACC synthase (ACS) and ACC oxidase (ACO), respectively. Few historical studies has documented the influence of temperature on ethylene biosynthesis, however. Biggs and Handa (1988) showed that the higher the temperature at which a tomato fruit is incubated, the lower its ACS and ACO activities, concordant with the observed failure to ripen (Yakir et al., 1984; Brandt et al., 2006). This study also found that ACS is more heat-sensitive than ACO. Similar heat-induced inhibition of ethylene biosynthesis was reported in kiwi fruit (Antunes and Sfakiotakis, 2000). Transcriptional analyses of *ACSs* and *ACOs* in tomato revealed the heat-stress induced expression of the *ACS3* in mature tomato pollen grains (Frank et al., 2009) and the upregulation of *ACO1*, *ACO4* in tomato leaf under heat stress condition (Pan et al., 2019). However, very little quantitative data is available linking gene expression of *ACO* and *ACS* during tomato fruit ripening to their protein abundance and their enzyme activity. Furthermore, much of effort have been conducted on heat sensitive tomato, while the ethylene biosynthesis from gene to protein and metabolite in heat tolerant tomato has not yet been profiled during fruit ripening.

Once produced, ethylene acts as a signaling molecule, binding to receptors (ETR), inhibiting the activation of Constitutive triple response (CTR), allowing the cleavage of C-terminal domain from ethylene-insensitive protein 2 (EIN2). The C-terminal part goes to the nucleus to activate ethylene responses via ethylene-insensitive 3/ethylene-insensitive3-like (EIL) family and ethylene response factors. Both ETRs and CTRs act as negative regulators, while EIN2 positively regulates the ethylene responses (McManus, 2012). A considerable amount of literature has been focused on the gene expression of ethylene signaling during fruit ripening of heat sensitive tomato cultivars. In general, *ETR3*, *ETR4*, and *ETR6* displays the peak in expression of at the onset of ripening, and *CTR1* shows a climacteric ripening-regulated expression during fruit ripening, while EIN2-mRNA level did not change during ripening (Kevany et al, 2008; Liu et al., 2015; Mata et al., 2018b). Mata et al. (2018a) revealed the feasibility of identification and absolute quantification of ethylene signaling proteins during tomato fruit ripening using mass spectrometry approach. However, studies about the proteomic quantification of ethylene signaling still receive limited attention and the ethylene signaling pathway of heat tolerant cultivars and the effect of high temperature on this pathway remains to be explored.

To overcome heat stress issues in food crops, a number of heat tolerant cultivars have been developed which can be grown under both mild and hot conditions (Alam et al., 1970; Hazra et al., 2007; Karkute et al., 2021). Tomato cv. Savior is a heat-tolerant cultivar which can be grown during both the Vietnamese winter and summer season (Tran et al., 2017). A previous phenotyping study of this cultivar showed that summer and winter fruit shared similar physiological and biochemical attributes including color, firmness, total soluble solid content and respiration rate, during on-vine ripening and postharvest storage (Tran et al., 2021). The role of the ethylene biosynthesis and signaling pathway during fruit ripening of classical heat sensitive tomato cultivars are well known and published (Adams and Yang, 1979; Xu and Zhang, 2015). However, it remains to be explored how this relates to the ripening regulation in a heat resistant cultivar. To this end, the regulations of ethylene biosynthesis and signaling in tomato during fruit ripening and postharvest storage of cv. Savior grown under Vietnamese winter and summer conditions were investigated. The measurements of ethylene production rate, metabolites ACC and MACC, and *in-vitro* activity of ACO and ACS were performed. Based on the previous study of absolute protein quantification using a targeted LC–MS based method (Mata et al., 2018a), we followed their approach to quantify all ETRs, CTRs and EIN2 and extend to measure the protein abundance of ethylene biosynthesis enzymes. To observe the correlation between transcript and protein levels, gene expression of the targeted proteins was investigated using real-time qPCR. Fruit quality attributes were analyzed to describe fruit phenotype during ripening and post-harvest storage. Our hypothesis is that the underlying ethylene biosynthesis and its signaling pathway of this heat tolerant tomato are identical to those of heat sensitive tomato cultivars but may differ quantitatively between winter and summer crop.

## 2 Materials and methods

### 2.1 Plant material

Tomato plants (*Solanum lycopersicum* L. cv. Savior) were grown in Bac Ninh, Vietnam from mid-November 2018 to March 2019 (winter), and from mid-February to June 2020 (summer). The range of average monthly temperature was from 18 – 24°C with the average rainfall of 34 mm in winter and from 21 – 32°C with the average rainfall of 67 mm in summer growing season (https://www.worldweatheronline.com/). Tomato fruit in those winter and summer seasons were harvested at the immature green (IMG), mature green (MG), breaker (BR), turning (TRN), orange (ORG), light red (LR) and red ripe (RR) stages. The maturity stages are based on the color of the tomato fruit and adapted from the Tomato expression atlas (https://tea.solgenomics.net/) (**Supplementary Table 1**). Harvested red ripe tomatoes were stored at 18°C and 80% RH and sampled after 6 d (RR + 6) or 12 d (RR + 12) to include postharvest storage.

### 2.2 Fruit quality measurement

Color (hue angle) was measured using a Minolta CM 2500d (Konica Minolta, Japan) according to Hertog et al. (2004). Firmness was defined as the maximum force (N) that an 8 mm cylindrical probe plunge experienced while indenting the fruit until a depth of 2 mm from the fruit surface at a velocity of 20 mm min^-1^. It was measured on three equidistant points on the equator of each tomato using a Mark-10- ESM 303 Texture Analyzer (USA). Total soluble solids contents (TSS) were determined for each fruit with a digital refractometer Atago PR-101 (Atago Co. Ltd., Japan) at room temperature. The acidity (TA) was determined by potentiometric titration with 0.1 mol L^-1^ NaOH up to pH 8.1, using 5 ml of diluted juice in 50 ml distilled water.

### 2.3 Measurement of respiration rate and ethylene production rate

The respiration rate (µmolCO_2_ kg^-1^ s^-1^) and ethylene production rate (nmol kg^-1^ s^-1^) were simultaneously measured using gas chromatography (GC) according to Bulens et al. (2011) with some modifications. Instead of measuring individual fruit, at each maturity or postharvest stage, a pool of four fruit was incubated in an airtight container for 2 h at 18°C. A 5 mL of gas sample was taken by a gas tight syringe and injected into a Clarus^®^580 GC (Perkin Elemer, USA). Afterward, pericarp tissue of each pool was flash frozen in liquid nitrogen, ground by a grindomixer (Retsch, Haan, Germany) and stored at -80°C for metabolite and molecular analysis.

### 2.4 Measurement of ethylene biosynthesis intermediates and enzymatic activity

1-Aminocyclopropane-1-carboxylic acid (ACC) quantification was performed using the method by Bulens et al. (2011) with some modifications. For the reaction step, 0.2 mL of the extract was diluted with 1.2 mL of distilled water. 1-(Malonylamino)cyclopropane-1-carboxylic acid (MACC) were measured as described by Bulens et al. (2011). ACO activity was measured according to the method of Van de Poel et al. (2012). ACS activity measurement was identical to that of Bulens et al. (2011). ACC and MACC concentration was expressed as µmol kg^-1^. ACS and ACO activity was expressed at nmol kg^-1^ s^-1^.

### 2.5 RNA extraction, reverse transcription and q PCR

Total RNA extraction and reverse transcription was performed according to Mata et al. (2018b). qPCR was performed using Bio-Rad CFX96 Touch Real-Time PCR Detection System with SsoAdvanced™ Universal SYBR^®^ Green Supermix (Bio-Rad, USA). The thermal cycling program was modified from SsoAdvanced™ Universal SYBR^®^ Green Supermix instruction manual (2013) with the polymerase activation and initial denaturation step at 95°C for 10 min, 40 cycles of denaturation at 95°C for 10 s, and annealing at 63°C for 20 s. To normalize the target genes, four housekeeping genes (HKGs) were selected: actin (*ACT*), elongation factor1 (*EF1*), glyceraldehyde-3-phosphate dehydrogenase (*GAPDH*) and 60S ribosomal protein L2 (*RPL2*). The expression stability of HKGs was tested using qbase and Bestkeeper. The primers used in this study were referenced from Van de Poel et al. (2012) and Mata et al. (2018a) (**Supplementary Table 2**). The specificity of each pair of primers was checked using the Basic Local Alignment Search Tool (BLAST) from NCBI (https://blast.ncbi.nlm.nih.gov/Blast.cgi) and its melting temperature was validated using the OligoAnalyzer™ Tool from IDT (https://eu.idtdna.com/calc/analyzer). The relative quantification of gene expression (dimensionless) was calculated according to Mata et al. (2018a).

### 2.6 Protein extraction, reduction, alkylation and in-gel digestion

Sample preparation was adapted from Mata et al. (2018b) with some modifications. After the first centrifugation, 4 mL of the total protein extract was concentrated using Amicon^®^ Ultra-4 Centrifugal Filter Units – 10,000 NMWL (Ireland) at 5000 x g for 50 min. The rest of the extract was ultra-centrifuged at 100,000 x g for 2 h at 4°C. The pellet containing membrane protein was resuspended in 100 – 500 µl of 10 mM Tris buffer (pH 7.5) containing 10% SDS. The total protein extract, the cytosolic protein extract, and the membrane protein extract was used for analyses of ACOs, ACSs and signaling proteins, respectively. All three types of the extract were fractioned using SDS-PAGE, the protein fragments ranging from 26 – 43 kDa (total protein) and from 55 – 180 kDa (membrane protein) were excised from the gel, cut into small pieces, de-stained and in-gel digested using sequencing grade modified trypsin (Promega, USA). After digestion, the peptide sample was desalted using Pierce^®^ C18 Spin Columns (ThermoFisher Scientific, USA) following the manufacturer’s instructions, and gently dried using Eppendorf Concentrator 5301. The peptide pellet was resuspended in 12 µL of buffer containing 2% acetonitrile and 0.1% formic acid. The peptide concentration was the highest absorbance recorded using a Nanodrop (ThermoFisher Scientific, USA) in the region of 190 – 316 nm.

### 2.7 Peptide identification and quantification

Peptide identification and protein quantification was performed using the parallel reaction monitoring (PRM) technique. The design of unlabeled and labeled synthetic peptides, the application of LC- tandem MS for identification and quantification was identical to Mata et al. (2018b) The list of unlabeled peptides is shown in **Supplementary Table 3**. The list of labeled peptides with known concentration is provided in **Supplementary Table 4**. The protein concentration of ACO and signaling receptors was expressed as µmol kg^-1^ total protein and µmol kg^-1^ membrane protein respectively.

### 2.8 Statistical analysis

To find significant differences between maturity stages, one-way ANOVA and Tukey’s multiple comparison were applied for ethylene production rate, and the non-parametric Kruskal-Wallis test was used for intermediates, enzymatic activities, and gene expression. Significant differences between two groups at one maturity stage were calculated using independent T-test. Given proteins quantified by a single peptide, Kruskal-Wallis comparison was used. For proteins expressed by more than one peptide, protein data were analyzed using the mixed model procedure according to Mata et al. (2018b). In this model, ‘maturity stage’ was considered as a fixed effect while ‘peptide’ was set as a random effect introducing a repeated structure ‘sample’ to account for the fact that the various peptides were covariates measured on the same fruit samples.

## 3 Results

### 3.1 Summer fruit show a delayed climacteric peak in ethylene production, yet ripened identical compared to winter fruit

To compare fruit phenotype during ripening and postharvest storage, physical and chemical properties of winter and summer tomato were measured (**Supplementary Figure 1**). Winter and summer fruit showed similar levels of TSS during ripening and postharvest storage, as well as the same rate of decline in hue and firmness. The acidity slightly decreased in winter fruit after LR, while it was relatively stable in summer fruit after increasing from IMG. Both winter and summer tomato showed a respiratory peak with its maximum at the TRN stage.

**Figure 1:**
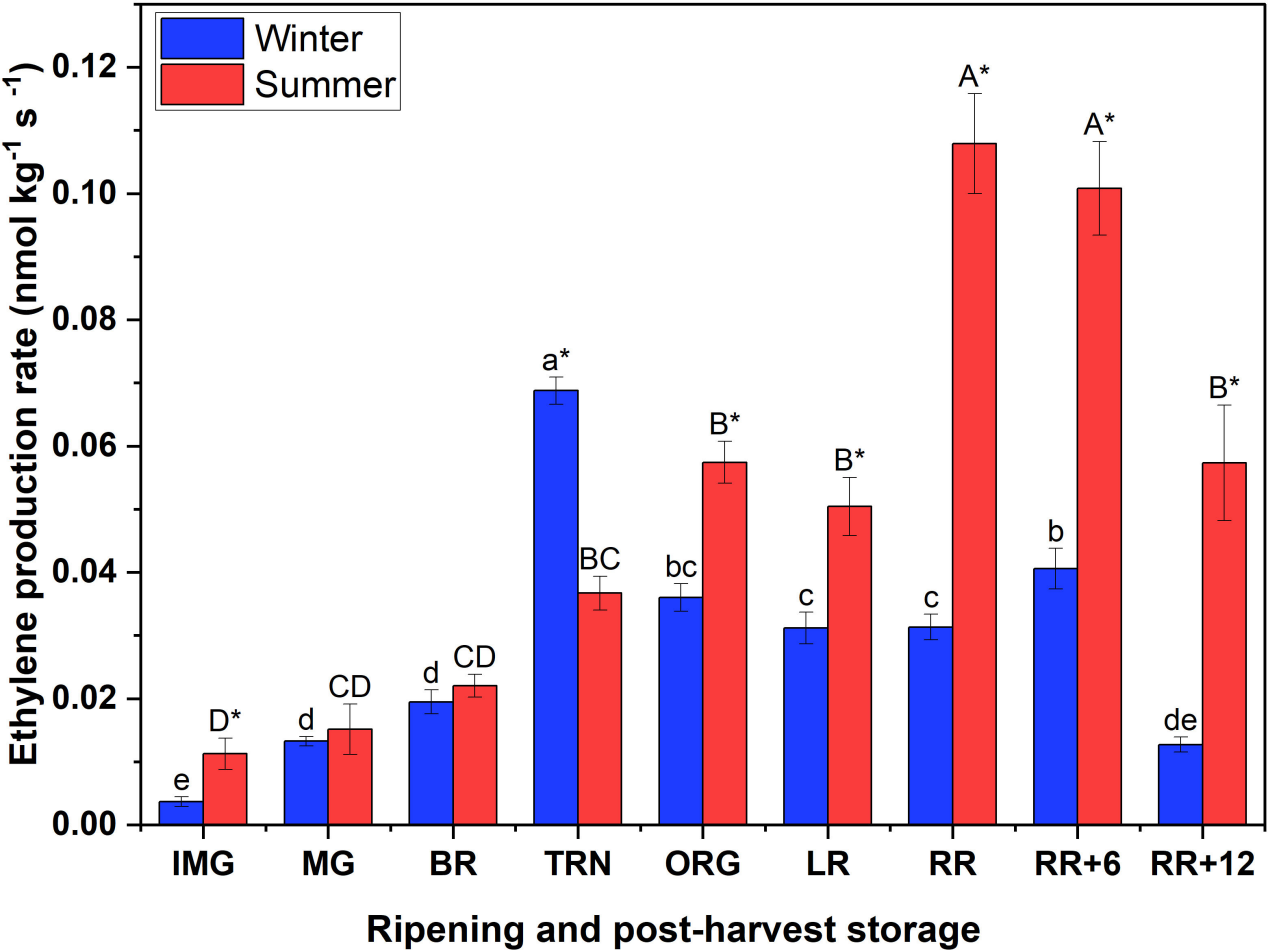
Ethylene production rate of winter and summer fruit during ripening and postharvest storage. IMG, immature green; MG, mature green; BR, breaker; TRN, turning; ORG, orange; LR, light red; RR, red ripe; RR+6, 6 d postharvest; RR+12, 12 d postharvest. Error bars represent the standard error of the mean (n = 5). Different lower letters show significant differences between stages of maturity and postharvest storage of winter fruit (p < 0.05), while different capital letters indicate significant differences between stages of maturity and postharvest storage of summer fruit (p < 0.05). Asterisk indicates significant differences between winter and summer fruit at each stage (p < 0.05).

The peak in ethylene production rate of winter fruit coincided with the respiratory maximum at TRN stage, while for summer fruit the ethylene peak was delayed until the RR stage (**Figure 1**). By the end of the storage period the ethylene production rate of winter fruit had dropped to the basal level, while the ethylene emission in summer fruit remained relatively high.

### 3.2 ACC, MACC and ACS/ACO activities were different between winter and summer fruit

**Figure 2:**
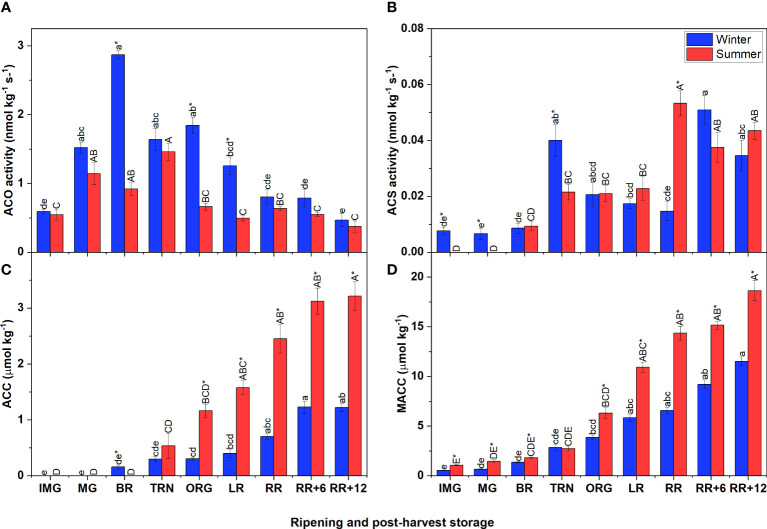
Ethylene biosynthesis metabolites and enzyme activities in winter and summer fruit during ripening and postharvest storage. **(A)** 1-aminocyclopropane-1-carboxylic acid oxidase (ACO) *in vitro* activity, **(B)** 1-aminocyclopropane-1-carboxylic acid synthase (ACS) *in vitro* activity, **(C)** ACC content, **(D)** MACC content in winter and summer fruit during ripening and postharvest storage. IMG, immature green; MG, mature green; BR, breaker; TRN, turning; ORG, orange; LR, light red; RR, red ripe; RR+6, 6 d postharvest; RR+12, 12 d postharvest. Error bars represent the standard error of the mean (n = 5). Different lower letters show significant differences between stages of maturity and postharvest storage of winter fruit (p < 0.05), while different capital letters indicate significant differences between stages of maturity and postharvest storage of summer fruit (p < 0.05). Asterisk indicates significant differences between winter and summer fruit at each stage (p < 0.05).

In both winter and summer fruit, the ACO activity followed a climacteric pattern which strongly increased when fruit started to ripen and then gradually decreased (**Figure 2A**). Particularly, the ACO activity of winter fruit increased sharply to peak at the BR stage, gradually declining afterwards. In summer fruit ACO activity reached its maximum only at the TRN stage. In both winter and summer fruit, these maximum values of ACO activity were reached one stage earlier compared to the corresponding ethylene climacteric peak. Like the ACO activity, the ACS activity increased during early fruit ripening followed by a period of decreasing (winter fruit) or constant (summer fruit) activity (**Figure 2B**). However, during postharvest storage, the ACS activity strongly increased again in both winter and summer fruit.

The initial levels of ACC, the precursor of ethylene biosynthesis, and MACC were very low but steadily increased throughout fruit ripening and postharvest storage (**Figures 2C, D**). Summer and winter fruit shared this same pattern, but the accumulation rate was higher in summer fruit. In both winter and summer fruit, the ACC content was overall lower than the MACC content.

### 3.3 Gene expression of ACO in summer fruit seems to be lower than those in winter fruit during ripening and postharvest storage

**Figure 3:**
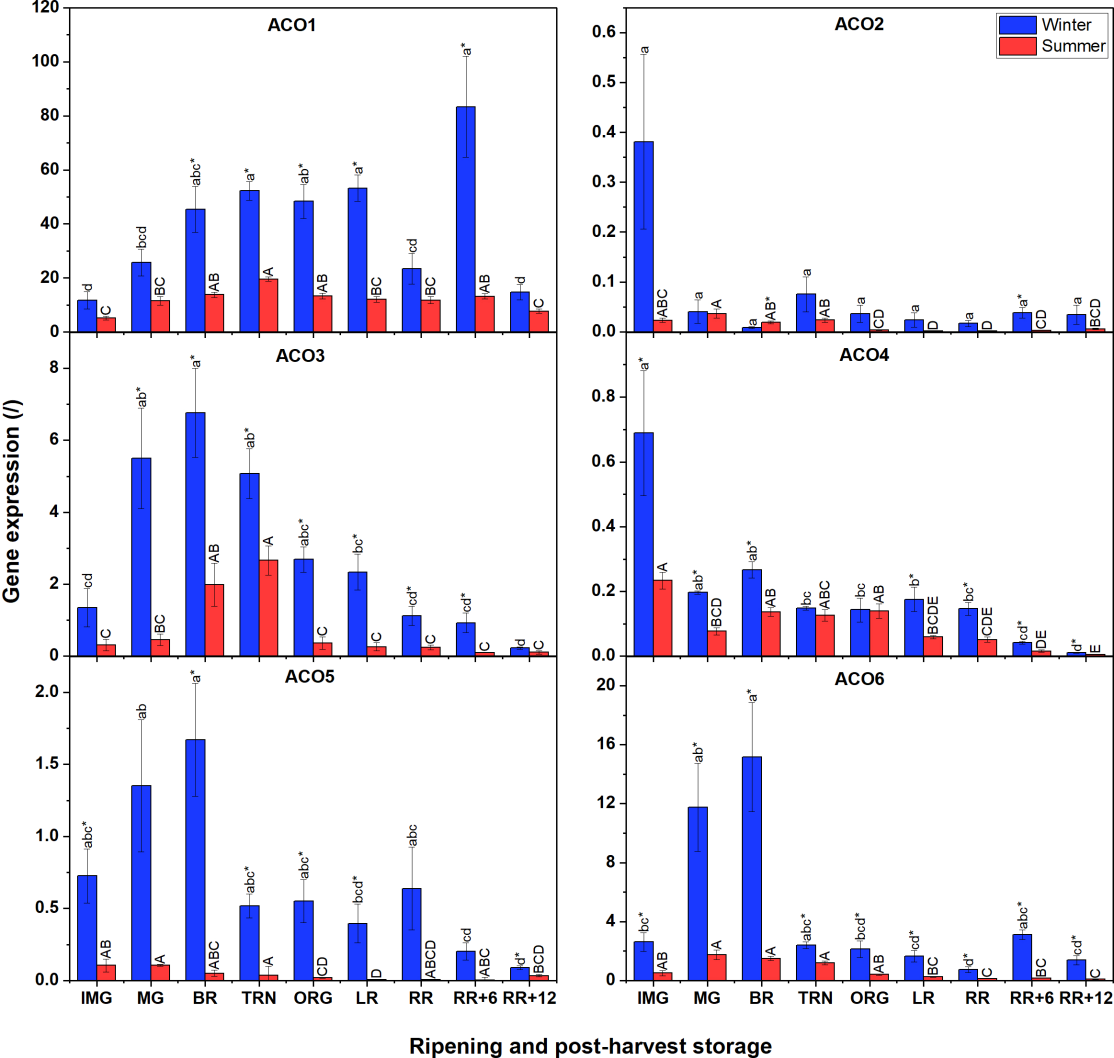
Gene expression of six ACO genes (ACO1-6) in winter and summer fruit during ripening and postharvest storage. IMG, immature green; MG, mature green; BR, breaker; TRN, turning; ORG, orange; LR, light red; RR, red ripe; RR+6, 6 d postharvest; RR+12, 12 d postharvest. Error bars represent the standard error of the mean (n = 5). Different lower letters show significant differences between stages of maturity and postharvest storage of winter fruit (p < 0.05), while different capital letters indicate significant differences between stages of maturity and postharvest storage of summer fruit (p < 0.05). Asterisk indicates significant differences between winter and summer fruit at each stage (p < 0.05).

Because the content of the ethylene biosynthesis intermediates and ACS/ACO activities were distinct between summer and winter fruit, we investigated if there were differences in gene expression of the *ACS* and *ACO* multigene family. We noticed that there were several similarities and differences in gene expression between winter and summer fruit (**Figure 3**). Gene expression analysis revealed that the expression of *ACO7* was undetectable in both winter and summer fruit. Overall, it is apparent that *ACO* gene expression was higher in winter fruit compared to summer fruit. In addition, in both winter and summer fruit, transcript levels of *ACO1*, *ACO3*, *ACO5* and *ACO6* were predominant, while *ACO4*, followed by *ACO2* were the least expressed. ACO1 had the highest expression level among ACO members in both winter and summer fruit, and highly expressed during ripening. The gene expression of *ACO3*, *ACO5*, and *ACO6* in winter fruit followed a climacteric pattern with their maximum reached at the MG and BR stages. A similar climacteric pattern was observed for *ACO3* in summer fruit but with a gradual increase from IMG to TRN. Both *ACO2* and *ACO4* expressions in winter fruit, highly induced at the IMG stage, rapidly fell to a very low expression level afterwards. The expression levels of *ACO2* and *ACO4* were largely comparable between summer fruit and winter fruit.

### 3.4 Gene expression of ACS in summer fruit seems to be lower than those in winter fruit during ripening and postharvest storage

**Figure 4:**
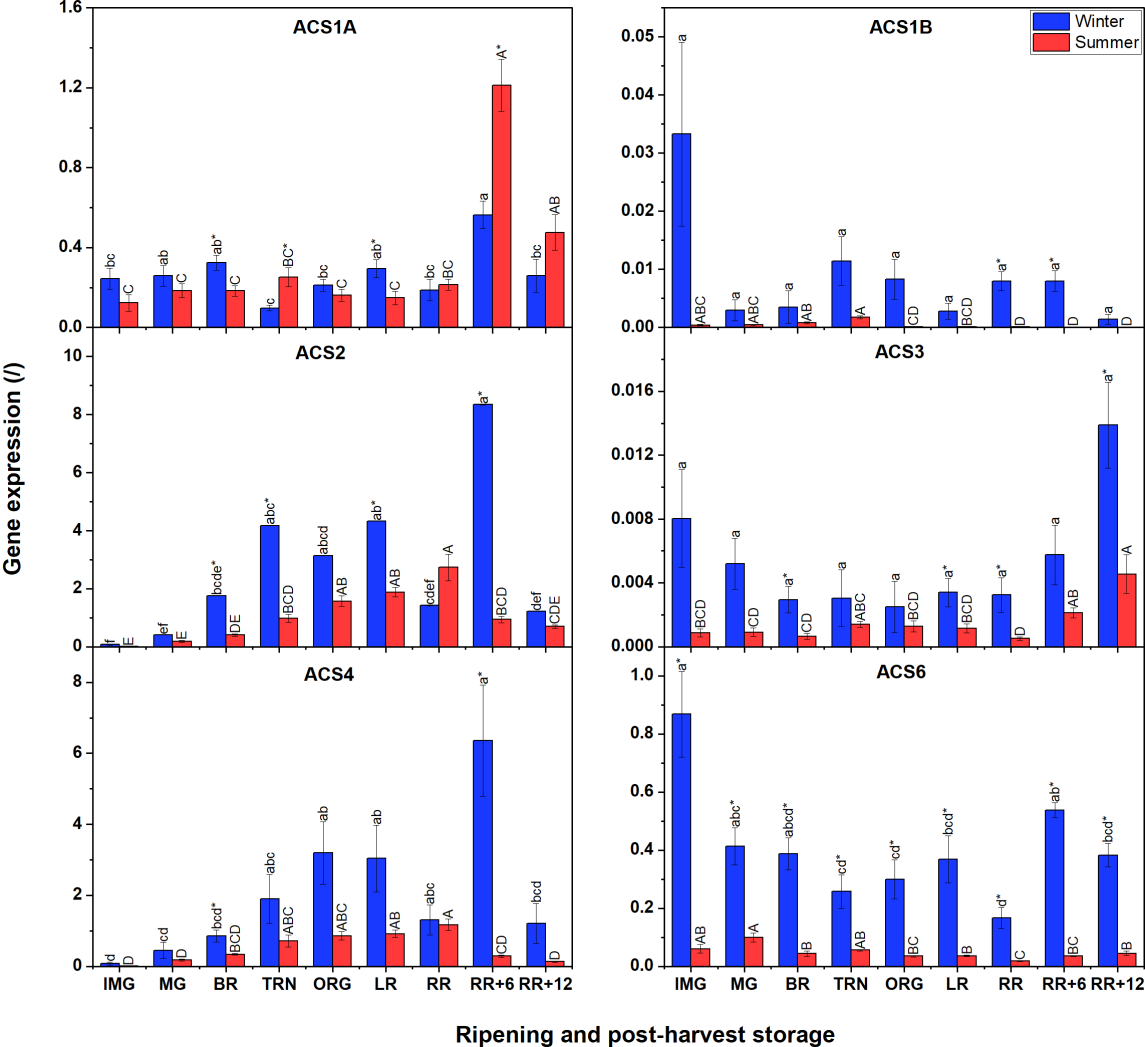
Gene expression of six ACS genes (ACS1A-B, ACS2-ACS4, ACS6) in winter and summer fruit during ripening and postharvest storage. IMG, immature green; MG, mature green; BR, breaker; TRN, turning; ORG, orange; LR, light red; RR, red ripe; RR+6, 6 d postharvest; RR+12, 12 d postharvest. Error bars represent standard error of the mean (n = 5). Different lower letters show significant differences between stages of maturity and postharvest storage of winter fruit (p < 0.05), while different capital letters indicate significant differences between stages of maturity and postharvest storage of summer fruit (p < 0.05). Asterisk indicates significant differences between winter and summer fruit at each stage (p < 0.05).

Compared to *ACO* gene expression, *ACS* transcript levels were generally lower (**Figure 4**). Additionally, winter fruit mostly showed higher *ACS* gene expression levels than summer fruit apart for ACS1A. No expression of *ACS5*, *ACS7* and *ACS8* was detected at all. In winter fruit, expression levels of *ACS2*, *ACS4* and *ACS6* were the highest, followed by *ACS1A*, *ACS1B*, and *ACS3*. Both *ACS2* and *ACS4* shared a similar expression pattern, which increased until the LR stage, subsequently dropped, to peak at the RR+6 stage before decreasing towards the final stage. *ACS6* was the most expressed *ACS* transcript. *ACS1A* expression gradually increased throughout fruit ripening, suddenly decreased at RR and recovered at RR+6 during postharvest storage, then decreased again. Both *ACS1B* and *ACS3* were faintly expressed during fruit ripening and postharvest storage. In summer fruit, *ACS6*-mRNA shared a similar expression profile to that in winter fruit. Meanwhile, the expression of *ACS2* and *ACS4* was gradually up-regulated during fruit ripening and peaked at the end stage of ripening. During postharvest storage, the expression of both two genes sharply decreased. *ACS1B* and *ACS3* were faintly expressed in both winter and summer fruit. *ACS1A* expression in summer fruit was somewhat different from that in winter fruit. Its expression steadily increased until RR, and subsequently decreased during postharvest storage.

### 3.5 Protein abundance of several ACO isoforms in winter and summer fruit are identical during ripening and postharvest storage

Protein identification was performed by the analysis of unlabeled synthetic peptides using unscheduled parallel reaction monitoring (PRM). Given *in silico* digestion from Skyline version 21.2 (Pino et al., 2020) and the shotgun data from Mata et al. (2018b) 91 unique peptides were representative for seven ACOs and nine ACSs. The uniqueness of each peptide was double checked against the tomato proteomic database using BLAST from Uniprot version 21.2 (Bateman, 2019). Using an unscheduled PRM approach, 49 unlabeled synthetic peptides were detected. In the pilot study, endogenous peptides from BR and TRN tomato were identified by comparison of the retention time, numbers of transitions and mass deviations to 49 detected synthetic peptides. ACO1 was identified based on two peptides, one with non-methionine-oxidative (non-Met(O)) and one with methionine-oxidative (Met(O)) modification. ACO5 was confirmed based on three peptides, and ACO6 with one peptide. The protein concentration of ACO1 was determined as the sum of non-methionine-oxidized and methionine-oxidized peptides. Endogenous peptides from ACO2, ACO4 and ACO7, and ACSs were not found. Given 99% of identity of ACO1 in exon 3, only one unique peptide of ACO3 in silico-digestion was found but we were unable to get it synthetized. However, this peptide was detected in Data Dependent Acquisition (**Supplementary Table 5**). The final concentration of ACO5 was calculated based on a mixed model approach. Individual peptide concentrations are shown in **Supplementary Figure 3**.

**Figure 5:**
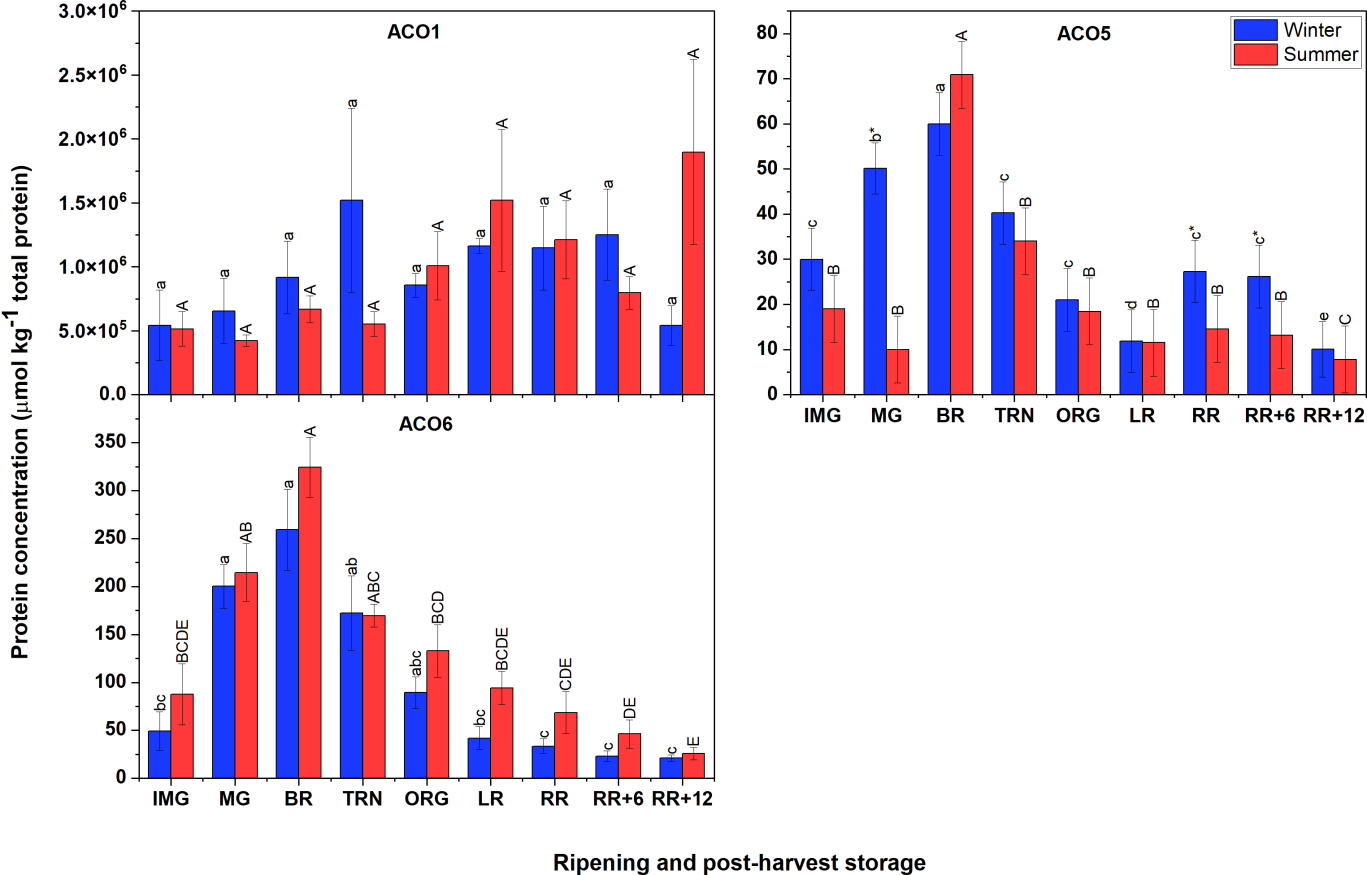
Protein concentration of ACO1, ACO5 and ACO6 in winter and summer fruit during ripening and postharvest storage. IMG, immature green; MG, mature green; BR, breaker; TRN, turning; ORG, orange; LR, light red; RR, red ripe; RR+6, 6 d postharvest; RR+12, 12 d postharvest. Error bars indicate standard error of the mean of ACO6 or standard error of the predicted mean of ACO1 and ACO5 (n = 3 in winter fruit and n =5 in summer fruit). Different lower letters show significant differences between stages of maturity and postharvest storage of winter fruit (p < 0.05), while different capital letters indicate significant differences between stages of maturity and postharvest storage of summer fruit (p < 0.05). Asterisk indicates significant differences between winter and summer fruit at each stage (p < 0.05).

**Figure 5** demonstrates that the most abundant proteins were ACO1, followed by ACO6, and finally ACO5. The finding also showed that there were no clear differences in protein abundance of ACO members between winter and summer fruit. In both winter and summer fruit, the protein abundance of ACO1 varied insignificantly. However, it is interesting to note that the protein concentration of ACO1 in the non-Met(O) form was about 1000 times higher than that of Met(O) ACO1 and they showed different patterns. In both winter and summer fruit, the protein abundance of Met(O) ACO1 gradually increased, peaked at RR stage, then subsequently declined (**Supplementary Figure 3**). In contrast, there was no significant variation observed for non-Met(O) ACO1. The protein abundance of ACO5 and ACO6 followed a climacteric pattern showing an increase early during ripening, and considerably decreased after the TRN stage in both winter and summer fruit.

### 3.6 Gene expression of receptors in summer fruit seems to be lower than those in winter fruit during ripening and postharvest storage

**Figure 6:**
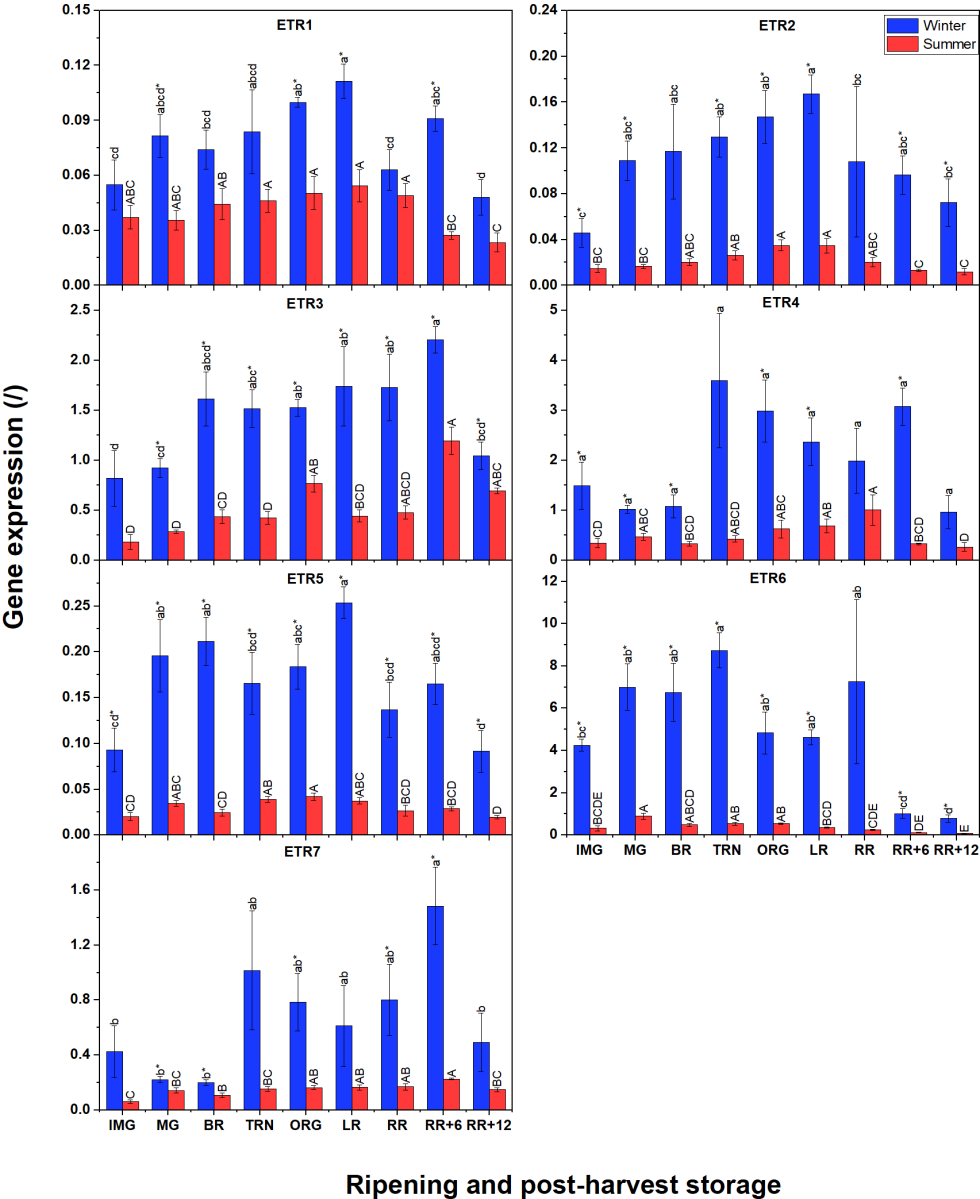
Gene expression of seven ethylene receptors (ETR1-7) in winter and summer fruit during ripening and postharvest storage. IMG, immature green; MG, mature green; BR, breaker; TRN, turning; ORG, orange; LR, light red; RR, red ripe; RR+6, 6 d postharvest; RR+12, 12 d postharvest. Error bars represent standard error of the mean (n = 5). Different lower letters show significant differences between stages of maturity and postharvest storage of winter fruit (p < 0.05), while different capital letters indicate significant differences between stages of maturity and postharvest storage of summer fruit (p < 0.05). Asterisk indicates significant differences between winter and summer fruit at each stage (p < 0.05).

Besides the biosynthesis pathway, we were also interested to know differences in ethylene signaling between winter and summer fruit. The gene expression profiles of six ethylene receptors (*ETR1*, *ETR2*, *ETR3*, *ETR5*, *ETR6*, and *ETR7*) are shown in **Figure 6**. Again, the expression of these receptors in winter fruit was much higher than in summer fruit. In both winter and summer fruit, transcripts of *ETR3*, *ETR4*, *ETR6* and *ETR7* were the most abundant while *ETR1*, *ETR2*, and *ETR5* were expressed at lower levels. The patterns of *ETR1*, *ETR2*, *ETR3* and *ETR5* transcripts in summer fruit were similar to those in winter fruit. For winter fruit, the expression of ETR6 was the highest and slightly increased until the TRN stage, to decrease again afterwards. Interestingly, *ETR4* and *ETR7* shared the same expression pattern, being initially at a low level to become highly expressed from the TRN stage until RR+6, and only to decrease at RR+12. In summer fruit, *ETR4* and *ETR7* expression gradually increased from the IMG to the RR stage. During postharvest storage of summer fruit, *ETR4* gene expression decreased to the basal level while the *ETR7* transcript level continuously rose until RR+6 before decreasing. The *ETR3*-mRNA pattern was also somewhat similar except that its level started to increase at the BR stage. *ETR1*, *ETR2*, *ETR5* were expressed at lower levels and shared a similar pattern showing a slight increase from the IMG to LR stage to decline afterwards.

### 3.7 Gene expression of CTRs, and EIN2 in summer fruit seems to be lower than winter fruit during ripening and postharvest storage

**Figure 7:**
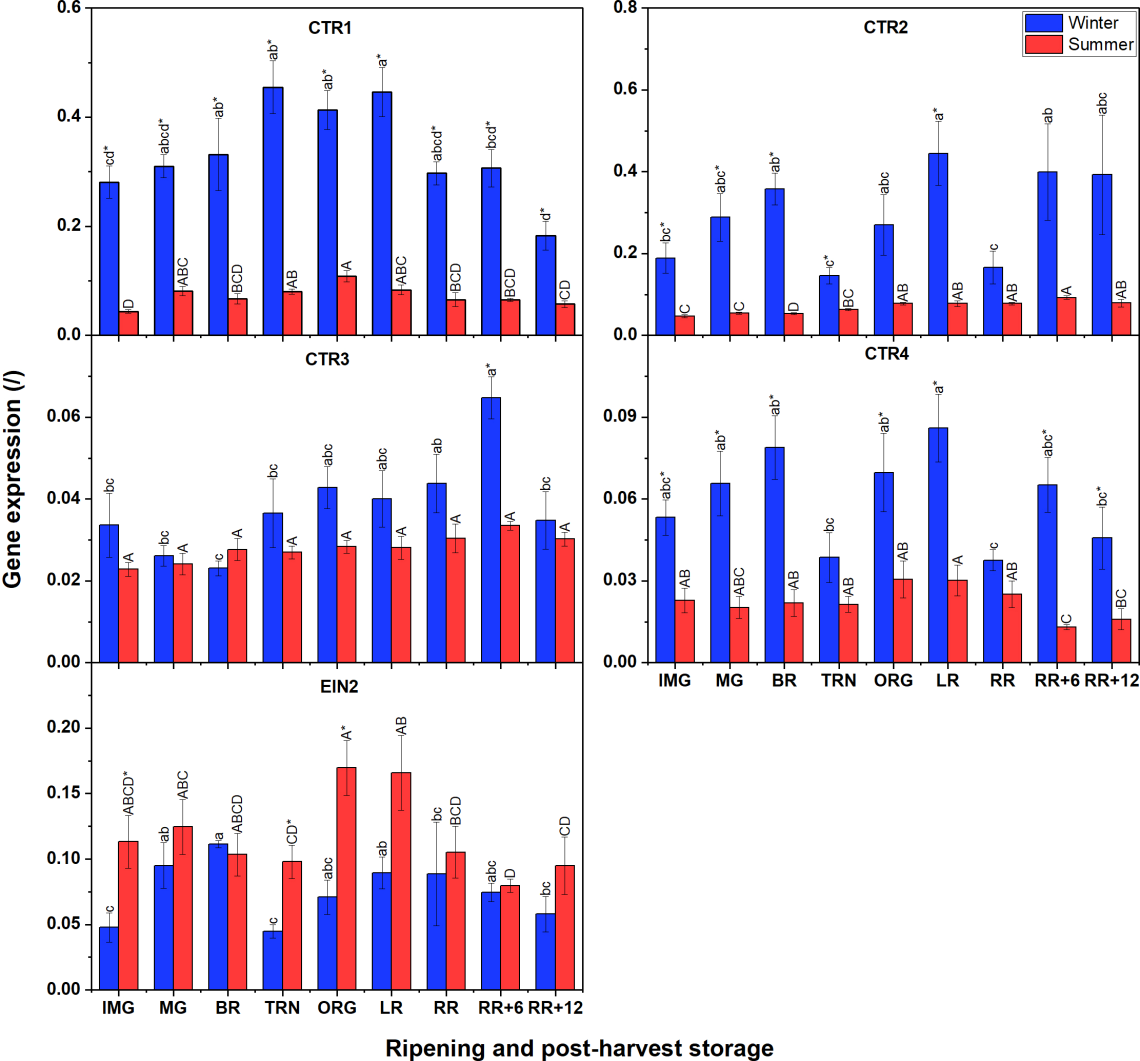
Gene expression of 4 CTRs (CTR1-4) and EIN2 in winter and summer fruit during ripening and postharvest storage. IMG, immature green; MG, mature green; BR, breaker; TRN, turning; ORG, orange; LR, light red; RR, red ripe; RR+6, 6 d postharvest; RR+12, 12 d postharvest. Error bars represent standard error of the mean (n = 5). Different lower letters show significant differences between stages of maturity and postharvest storage of winter fruit (p < 0.05), while different capital letters indicate significant differences between stages of maturity and postharvest storage of summer fruit (p < 0.05). Asterisk indicates significant differences between winter and summer fruit at each stage (p < 0.05).

Unlike for *ETR* gene expression, not all *CTR* expression in winter fruit was significantly higher than that in summer fruit (**Figure 7**); only for *CTR1*, *CTR2* and *CTR4* this was the case. Among the four *CTRs*, *CTR1* and *CTR2* were the highest expressed, followed by *CTR4* with *CTR3* being the least expressed. A climacteric behavior was observed for *CTR1* expression in tomato grown in both seasons which increased until the TRN stage, remained constant until LR, to decrease afterwards. Prominently, *CTR2* and *CTR4* in winter fruit shared the same fluctuating pattern of gene expression throughout the whole experiment. *CTR3* was clearly expressed before ripening, reached its peak after 6 d of postharvest storage and then decreased again. In summer fruit, both *CTR1* and *CTR4*, and also *CTR2* and *CTR3*, shared similar expression patterns. For *EIN2*, its general expression was not distinctly different between winter and summer fruit. In winter fruit the expression of *EIN2* slowly increased before and at the beginning of ripening stages, then strongly decreased at the TRN stage, and recovered afterwards. In summer fruit, the *EIN2* transcript was expressed at a rather low level and it strongly decreased from the BR to the TRN stage, then recovered gradually at later stages. There was a slight decline of its expression throughout postharvest storage which was observed in both winter and summer fruit.

### 3.8 Protein abundance of receptors, CTRs, and EIN2 in winter and summer fruit are identical during ripening and postharvest storage

To quantify 12 signaling proteins, 21 unique peptides were used similar to Mata et al. (2018b) (**Supplementary Table 4**). ETR1, ETR2, and EIN2 were each identified by one peptide; ETR3, ETR6, ETR7, CTR1 and EIN2 with 2 peptides; ETR4 with 3 peptides. Endogenous peptides of ETR5, and CTR3, and CTR4 were not found. For those proteins identified by more than one peptide, the protein abundance was represented based on a mixed model approach. Individual peptide concentrations are shown in **Supplementary Figure 4.**

**Figure 8:**
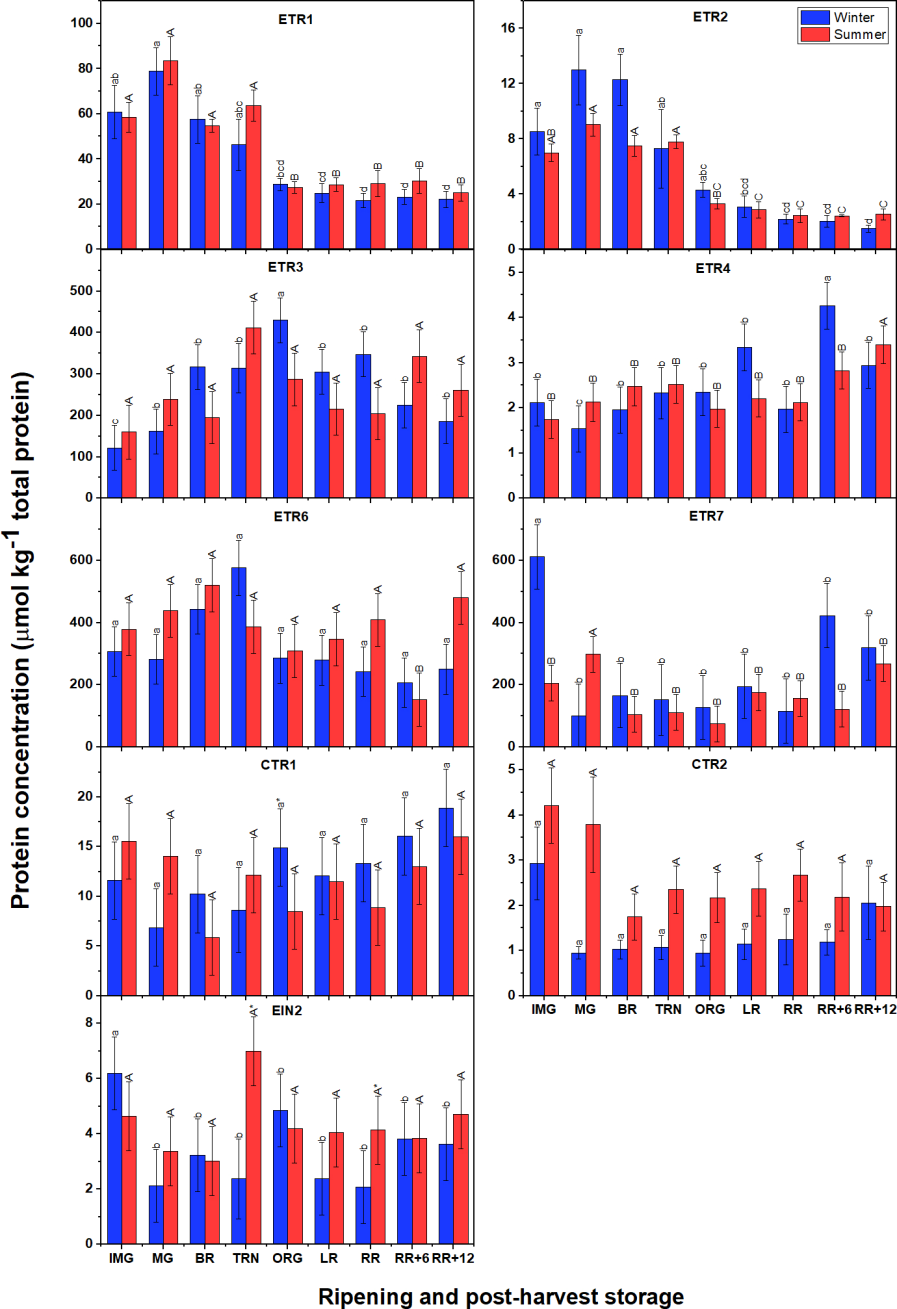
Protein concentration of six ethylene receptors (ETR1-4, ETR6-7), CTR1, CTR2 and EIN2 in winter and summer fruit during ripening and postharvest storage. IMG, immature green; MG, mature green; BR, breaker; TRN, turning; ORG, orange; LR, light red; RR, red ripe; RR+6, 6 d postharvest; RR+12, 12 d postharvest. Error bars indicate standard error of the mean (n = 5). Different lower letters show significant differences between stages of maturity and postharvest storage of winter fruit (p < 0.05), while different capital letters indicate significant differences between stages of maturity and postharvest storage of summer fruit (p < 0.05). Asterisk indicates significant differences between winter and summer fruit at each stage (p < 0.05).

Surprisingly, the signaling proteins in summer fruit were present at levels comparable to those observed in winter fruit (**Figure 8**), which is very distinct from the gene expression profiles. Among the receptors, ETR3, ETR6 and ETR7 were the most abundant proteins, followed by ETR1, and finally ETR4. Both ETR1 and ETR2 showed similar patterns with their abundance peaking around the MG stage to subsequently decrease towards the ORG stage and remain at low levels afterwards. In winter fruit, the gradual accumulation of ETR3 protein was observed from IMG to ORG stage then slightly reduced. A similar behavior of ETR3 protein level was observed in summer fruit but remaining insignificantly different between stages. A distinct behavior of ETR4 abundance was observed in which there were no significant changes from the IMG to the RR stage after which a slight increase was observed during postharvest storage. In general, the ETR6 protein pattern was quite similar to ETR3 but no remarkable differences were observed. Unlike other receptors, ETR7 was highly abundantly before and at the onset of ripening, then its abundance strongly declined and maintained relatively constant during ripening and postharvest storage. In terms of CTR proteins, CTR1 abundance was higher than CTR2. Both CTR proteins retained their level throughout fruit ripening and postharvest storage. EIN2 also shared insignificant changes throughout fruit ripening and postharvest storage in both winter and summer fruit.

## 4 Discussion

### 4.1 Ethylene biosynthesis differs between tomato grown in winter and summer

Heat tolerant cultivars have been developed with desired traits and optimal responses to heat stress. Tomato cv. Savior (Syngenta AG, Switzerland), a heat-tolerant cultivar, was proven to be able to grow during the Vietnamese summer with high yield performance and good appearance (Genova et al., 2013). In this study, like many others, fruit were sampled based on color stage and represented accordingly. As a result, the figures do not provide information on the possible difference in rate of ripening during the winter or summer period as a function of calendar time. Winter and summer fruit exhibited similar respiration rate patterns, showing that once triggered at the onset of ripening, the respiration follows a typical climacteric rise regardless of the ethylene pattern. The acidity of the fruit is a contributor to the flavor of the tomato (Barrett et al, 2010). The high acidity in summer fruit indicates that summer fruit is sourer than winter fruit. Altogether our observations indicate that summer fruit can ripen as normally as winter fruit without clear differences in phenotype except for sourness. Little is known about the influence of growing season on ethylene biosynthesis and its signaling during fruit ripening and postharvest storage. This study revealed that the ethylene production rate, ACO and ACS activities, and the accumulation of ACC and MACC content in winter (18 – 24°C) and summer (21 – 32°C) were quite different during fruit ripening and postharvest storage. Van de Poel et al. (2012) reported ethylene production rate of tomato fruit was related to the S-adenosyl methionine (SAM) content. It can be hypothesized that the high ethylene production in summer fruit is due to a putative up-regulation of SAM levels and subsequent ACC accumulation. In winter fruit, the ethylene production rate quickly dropped to basal levels after reaching its climacteric peak which is in line with other studies (Giovannoni, 2004; Oms-Oliu et al., 2011; Van de Poel et al., 2012). However, we did not observe this clear drop in summer fruit, where the ethylene production rate remained relatively high at the end of the storage period. The enzyme analyses suggests that, in winter fruit, ACS is the rate limiting enzyme mainly during ripening, while during postharvest storage, ethylene production is rather controlled by ACO. This is supported by previous observations on ACO being rate limiting under certain conditions such as postharvest storage (Muench et al, 2012; Van de Poel et al., 2012; Grierson, 2014). In summer fruit however, ethylene production paralleled ACS *invitro* activity both during ripening and postharvest storage, suggesting that ACS rather than ACO is the rate limiting enzyme. Low ACS activity could be due to a low protein abundance or to not all of the ACS proteins being activated (Kim and Yang, 1992). Biggs and Handa (1988) reported a reduced activity of ACO and ACS under elevated temperatures, resulting in the inhibition of ethylene production. A similar tendency was observed in kiwi fruit (Antunes and Sfakiotakis, 2000). In contrast to these findings, heat did not as severely suppress the activities of the two key enzymes, and the ethylene production rate which might explain the heat tolerance of ‘Savior’ tomato. Consistent with literature, the continuous buildup of MACC to levels much higher than ACC, confirmed that MACC is an alternative end product diverting ACC away from the ethylene biosynthesis pathway (Peiser and Yang, 1998; Van de Poel et al., 2012). Collectively, our results seem to suggest that ACC production by ACS and concomitant conjugation towards MACC was higher in summer fruit, while winter fruit were faster in converting ACC into ethylene by ACO and therefore showed an earlier climacteric peak in ethylene synthesis.

### 4.2 Gene expression, protein abundance and enzymatic activity of ethylene biosynthesis in winter and summer

Quantitative RT-PCR analysis showed gene expression of *ACOs* in winter fruit to be much higher than that in summer fruit. A similar tendency was observed for *ACS*. However, for our experiments, the mean cycle threshold (Ct) values of the four selected HKGs of winter fruit were approximately three times higher than those of summer fruit (**Supplementary Figure 1**). This might explain the apparent high gene expression of the genes of interest in winter fruit compared to summer fruit, as they were expressed relative to the HKGs. Based on literature *ACT*, *EF1*, *GAPDH* and *RPL2* were chosen as being four classical HKGs used to normalize gene expression in tomato fruit during fruit development, ripening, and postharvest storage as well as under different gas treatments (Van de Poel et al., 2012; Mata et al., 2018b). *EF1* was also used as reference gene in tomato anthers under heat stress condition (Giorno et al., 2010). Our study found that the expression of the four selected HKGs was stable during fruit ripening and postharvest storage but differed between winter and summer fruit, reflecting the seasonal impact of, probably, heat on HKGs.

Gene expression is known to be controlled by various transcription factors. In tomato, it was found that HB-1 (a class-I homeodomain leucine zipper (HD-Zip) protein), NAC (No Apical Meristem, ATAF, Cup-shaped Cotyledon), ERF2 (ethylene response factor2) and a homolog allele TERF2 are transcription factors positively regulating *ACO1*, *ACO3*, and *ACO4* (Lin et al., 2008; Klee and Giovannoni, 2011), while RIN negatively regulates *ACS1A*, *2*, *4*, *6* (Martel et al., 2011; Dong et al., 2013). However, little is known about the heat sensitivity of these transcription factors. Furthermore, post-transcriptional regulation also plays a role in the regulation of mRNA levels, including destabilization by non-coding RNAs (ncRNAs) or degradation. There has been no detailed investigation on the post-transcriptional regulation of ACO and ACS members. Zuo et al. (2020) revealed that *ACS4*, *ACO2*, *CTR1* are potentially targeted by several microRNAs, long non-coding RNAs and circle RNAs in tomato fruit. In addition, *microRNA1917*, an miRNA down-regulating *CTR4* transcript level, was reported to up-regulate *ACS2*, *ACS4*, *ACO1* and *ACO3* transcripts from IMG through the RR stage (Wang et al., 2018; T. Yang et al., 2020). In our study, the decrease in *CTR4* expression at RR stage and the increase in *ACO1*, *ACS2* and *ACS4* expression at RR+6 in winter fruit may suggest the post-transcriptional regulation of *microRNA1917*. However, the decrease of *ACO1*, *ACS2*, *ACS4* during postharvest storage, which was in line with that of *CTR4*, suggests that *ACO* and *ACS* mRNAs in summer fruit may be post-transcriptionally regulated by other mechanisms such as destabilization by microRNAs (Wu et al., 2019). The difference in *ACO* and *ACS* gene expression between winter and summer fruit evokes the possibility that heat may alter the activation of transcription factors of *ACOs* and *ACSs* and their post-transcriptional regulation. In both winter and summer fruit, the expression of *ACS* genes was lower than that of *ACO*. This suggests a higher induction of transcription factors for *ACO* or a lower *ACO*-mRNA degradation as compared to *ACS*. Our expression analysis of *ACSs* suggests that *ACS2*, *ACS4* and *ACS6* are the main ACS genes expressed in both winter and summer fruit, which was in agreement with Van de Poel et al. (2012). The mismatch between *ACS* gene expression and ACS activity corroborates the possibility that ACS proteins are regulated by post-translational modifications such as phosphorylation, and homo/heterodimerization (Park et al, 2021). To clarify the contribution of each ACS member for enzymatic activity, the proteomic analysis should be further investigated.

Low peptide levels might either result from low expression levels or uncontrolled degradation during the extraction process. As we used the complete EDTA-free protease inhibitor cocktail to prevent the degradation of the extracted proteins, the low protein concentration that made the ACS, and additionally ACO2, ACO4 and ACO7, peptides undetectable must have been due to their low expression levels. The protein analysis showed no remarkable differences in protein abundances between winter and summer fruit in spite of the *ACO* transcripts being higher in winter fruit. As previously explained, the apparent difference in *ACO* gene expression between winter and summer fruit was likely affected by the different *HKGs* expression levels. In this research, the patterns of gene expression of *ACO5* and *ACO6* in winter fruit are in agreement with their protein levels, while in summer fruit, only the ACO6 protein abundance highly correlates with its gene expression. In both winter and summer fruit, a weak correlation was observed between *ACO1* gene expression and its protein level because of the insignificant difference in ACO1 protein abundance. (**Supplementary Figure 6**). The lack of difference in protein abundance of ACOs between winter and summer fruit might suggest the involvement of some protection mechanism through for instance heat shock proteins (HSPs). They facilitate to refold and stabilize protein, preventing dysfunctional protein conformation (Vierling, 1991). Karkute et al. (2021) reported that under heat stress, gene expression of many heat shock transcript factors and *HSPs* were up-regulated in two heat tolerant tomato genotypes, H88-78-1 and CLN-1621. However, until recently, no research has been done on their specificity to particular target proteins. The similarity between winter and summer fruit at the protein level suggests that high temperature had no major influence on the ACO translation but the activation of HSPs.

Among ACO members, ACO1 proteins were the most abundant, followed by ACO6 and ACO5 which is consistent with their transcript levels indicating that these are the main isoforms contributing to ACO activity. With respect to ACO1, the abundance of non-Met(O) peptide predominantly contributed to the total ACO1 abundance rather than the Met(O) peptide (**Supplementary Figure 4**). The exact function of the oxidative modification of ACO1 remains unknown. *ACO1* transcript level and total protein abundance were not well correlated to overall ACO activity. On the other hand, the pattern of ACO5 and ACO6 in terms of both gene expression and protein abundance correlates to some extent with the overall ACO activity (**Supplementary Figure 6**). This suggest that the ACO is predominantly controlled by ACO5 and ACO6 rather than by ACO1. As mentioned before, we were unable to synthesize the unique peptide for ACO3 and therefore were not able to quantify it. However, given the peptide was detected using DDA approach (**Supplementary Table 5**) and given its relatively high gene expression we should expect ACO3 to still substantially contribute to the overall ACO abundancy and its activity.

### 4.3 Quantitative difference in ethylene signaling between winter and summer fruit during ripening and postharvest storage

Given the lack of quantitative data on ethylene signaling proteins of tomato under heat stress conditions, the present study was designed to understand the regulation of the ethylene signaling pathway from mRNA to protein. Gene expression of six ethylene receptors (ETRs) and four CTRs was higher in winter fruit than in summer fruit, although there were no differences in protein abundance. Part of this might reflect the difference in the transcription of HKGs which were used to calculate the relative expression of our genes of interest.

We found that both in winter and summer fruit, ETR3, ETR4, ETR6, and ETR7 were the most expressed receptor transcripts during fruit ripening; this supports evidence from previous observations (Liu et al., 2015; Chen et al., 2018; Mata et al., 2018b). Another important finding was that the mRNA levels of ETR4, ETR7 in winter fruit, and of ETR3, ETR7 in summer fruit highly correlated with ethylene production rate during fruit ripening and postharvest storage (**Supplementary Figure 7**). These findings partially match with that of Kevany et al. (2007) who found a significant increase in ETR3, ETR4 and ETR6 transcripts when fruit started to ripen. However, Mata et al. (2018b) showed that only ETR3 transcripts changed significantly throughout fruit ripening. It is, thus, plausible that different tomato cultivars, or one cultivar grown in different seasons, can have a distinct ETR gene expression profiles. Differences in gene expression pattern between winter and summer tomatoes may due to the involvement of transcription factors and post-transcriptional regulation as mentioned earlier. Data analysis of protein quantification showed that in both winter and summer tomato, ETR3, ETR6 and ETR7 were the most abundant proteins in line with their transcript levels. For ETR4, although its gene expression was high, its protein abundance was low suggesting that its translation efficiency was low. These findings are also in line with Mata et al. (2018b). It is surprising that the ETR1 protein was highly abundant while its transcript level was the lowest among the 7 receptors studied in both winter and summer fruit. This outcome is in contrast to that of Mata et al. (2018b) who found both transcript and protein levels of ETR1 to be low. This suggests that the contribution of ETR members to signal transmission is different between cultivars. In both winter and summer fruit, ETR transcript and protein levels were not proportional, which support previous research, indicating post-translational regulation of ETR proteins such as complex formation and phosphorylation. The decline in protein abundance of ETR1 and ETR2, the climacteric protein pattern of ETR3, and the steady protein levels of ETR6 and ETR7 during ripening observed in the current study were also reported by Mata et al. (2018b). Several reports have shown an increase in ETR4 protein level at the onset of ripening (Kamiyoshihara et al., 2012; Mata et al., 2018b). This does not appear to be the case in our study in which the ETR4 protein abundance remained constant throughout fruit ripening and postharvest storage in both winter and summer tomato. In tomato, ETR1, ETR2 and ETR3 belong to subfamily I, whereas ETR4, ETR5, ETR6, and ETR7 belong to subfamily II (Chen et al., 2018). It has been observed that transgenic tomato in which ETR4 or ETR6 is silenced increase ethylene sensitivity, while suppression of ETR1 or ETR3 decrease ethylene sensitivity (Lanahan et al., 1994; Kevany et al., 2007; Kevany, et al., 2008; Okabe et al., 2011). In 2005, O’Malley et al., 2005 found that the ethylene binding activity of ETR isoforms in tomato is not identical, in which ETR3 showed the highest levels of ethylene binding, following by ETR1. Besides, heteromeric interactions of ETRs have been addressed recently by Kamiyoshihara et al. (2022) revealing that ETR4 in subfamily II forms heterodimers with subfamily I receptors (ETR1, ETR2, ETR3). Together, these findings suggest that ethylene binding affinity can be controlled by hetero-protein complexes between ETR4 (in subfamily II) and subfamily I ETRs and vice versa, subsequently controlling the ethylene sensitivity. It is, therefore, possible that the increase in ETR3 protein abundance, together with the high ETR1 and ETR2 protein abundance in our data may allow more ethylene binding and increase ethylene sensitivity. On the other hand, the drop in ETR1 and ETR2 protein concentration after the TRN stage and the slight decrease in ETR3 after the ORG stage may reduce ethylene sensitivity. Further studies are needed to understand the heteromeric complex formation between other subfamily II ETRs, (ETR5, ETR6, ETR7) and subfamily I ETRs. Finally, phosphorylation is also important to regulate ETR stability and activity as Kamiyoshihara et al. (2022) found that the phosphorylation level of ETR3 and ETR4 in tomato was distinct during fruit ripening and under various gas treatment conditions.

We also found that the protein abundance of CTR1 was the highest in both winter and summer fruit, followed by CTR2. This is in line with earlier observations (Liu et al., 2015; Mata et al. 2018b) indicating that CTR1 is the main negative regulator of ethylene signaling. No protein abundance was found for CTR3 and CTR4 which can be explained by their very low transcript levels (**Figure 7**). *CTR1* gene expression correlated with ethylene production rate in winter fruit, but not in summer fruit (**Supplementary Figure 8**). In addition, CTR1 protein abundance was similar between winter and summer fruit, which weakly correlated to ethylene production rate. These observations indicate that CTR1 transcription is up-regulated by ethylene but translation is not.

A qualitative study by Mata et al. (2018b) found that both EIN2 gene expression and its protein concentration gradually declined during ripening. Our results also showed a decline in EIN2-mRNA level during ripening and postharvest storage in both winter and summer fruit. Poor correlations between EIN2 gene expression, protein level and ethylene production rate indicate that the transcription of EIN2 is not induced by ethylene. The EIN2 protein abundance, however, was stable throughout ripening and postharvest storage. Ethylene binding to ETRs leads to less phosphorylation of EIN2 by CTR, resulting in the cleavage of the EIN2 C-terminal part (Binder, 2020). Based on the alignment of EIN2 between *Arabidopsis* and tomato using Uniport, Mata et al. (2018b) showed that the first peptide of EIN2 identified in tomato may include the cleavage location, while the second peptide identified appear to be in the C-terminal domain. However, given the membrane protein extraction method applied, the cytosolic EIN2 C-terminal portion should be excluded from the analyses. Hence, the measured EIN2 protein data represents the intact EIN2 protein concentration only. It is suggested that the stable EIN2 concentration is the consequence of the balance between translation, degradation and EIN2 C-terminal cleavage. Given the three times higher expression of HKG in summer compared to winter fruit, the absolute *EIN2* gene expression in summer fruit is higher than that in winter fruit, while there was no difference in EIN2 protein abundance between winter and summer fruit. This might indicate an initial translational regulation of EIN2 by stress granules (SPs) under heat stress. SPs, membraneless assemblies of mRNA and protein (RNP), are well-known to appear and stall the translation initiation when plant cells are exposed to stress conditions such as hypoxia or heat stress (Nguyen et al., 2016; Jang et al, 2020). As mentioned earlier, the lack of differences in protein abundance of ETR, CTR members and EIN2 also suggests the activation of HSPs to prevent dysfunctional protein conformation under heat stress condition.

## 5 Conclusion

The current study contributes to our understanding of regulation in ethylene biosynthesis and its signaling and the impact of growing season on fruit ripening in a heat tolerant tomato cultivar. We characterized the ethylene biosynthesis and its signal transmission in the heat tolerant tomato cv. Savior by physiological and gene expression and protein analysis as affected by seasonal conditions (Vietnamese winter versus summer). Winter fruit displayed a typical climacteric behavior of ethylene production rate, while summer fruit showed a delayed peak of ethylene production until the red ripe stage. The results of enzymatic activities, and their protein abundance between winter and summer fruit suggest that the ACO activity is mainly controlled by ACO5 and ACO6 rather than by ACO1. Likewise, ETR1, ETR3, ETR6, and ETR7 together with CTR1 largely control ethylene signal transduction. Different mRNA levels between winter and summer fruit are due to the expression of four selected HKGs which appears to be affected by heat stress. Different patterns of gene expression between winter and summer fruit suggest that heat stress might impact on the transcription factors and the post-transcriptional regulation. Similarities in protein abundance suggests the involvement of translation and post-translational regulation and possibly heat shock proteins. To have a wide-ranging overview of the regulation of these two pathways in heat tolerant cultivars, more research is suggested. This study is limited by the lack of information on the abundance of ACO3 and all ACS members. Consequently, it is unclear whether ACO3 is a main isoform and which ACS members are important driving factors of ACS activity. In ethylene signaling, the turnover of ETR, CTR members and EIN2 C-terminal part remain unknown. To achieve a more quantitative approach of the integrated picture of the regulation of fruit ripening under heat stress condition, a kinetic modelling study is currently being performed.

## Data availability statement

The data presented in the study are included in the supplementary material, further inquiries can be directed to the corresponding author.

## Author contributions

TN executed the experiments, analyzed the data and wrote the first draft of the manuscript. MH, DT and BN contributed to conception and design of the study. MH contributed to the statistical analysis. All authors contributed to the article and approved the submitted version.
