# Supplementary material for: Targeted system approach to ethylene biosynthesis and signaling of a heat tolerant tomato cultivar; the impact of growing season on fruit ripening
Source: Front Plant Sci. 2023 Jun 30;14:1195020. doi: 10.3389/fpls.2023.1195020 (PMC10348052; doi:10.3389/fpls.2023.1195020)
Supplement: Supplementary file 1 [file DataSheet_1.docx]

**Supplementary Fig. 1.** Respiration rate, hue and firmness in winter and summer tomato at IMG, during ripening and post-harvest storage. Error bars indicate standard error of the mean (n = 5). Different lower letters show significant difference between maturity stages and post-harvest storage in winter or summer tomato. Different capital letters show significant difference between winter and summer tomato at each stage.

**Supplementary Fig. 3.** Ct values of 4 housekeeping genes in winter and summer fruit under different conditions (on-vine ripening, 1-MCP and Ethylene treatment)

**Supplementary Fig. 4.** Absolute quantification of the individual peptides of ACO isoform containing more than two peptides in winter and summer fruit during ripening and post-harvest storage. Error bars indicate standard error of the mean (n = 3 in winter fruit and n =5 in summer fruit).

**Supplementary Fig. 5.** Absolute quantification of the individual peptides of signaling proteins containing more than two peptides in winter and summer fruit during ripening and post-harvest storage. Error bars indicate standard error of the mean (n = 3 in winter fruit and n =5 in summer fruit).

**Supplementary Fig. 6.** Correlation between ACO gene expression, its protein abundance and its enzymatic activity in winter (blue) and summer fruit (red) during ripening and post-harvest storage.

**Supplementary Fig. 7.** Correlation between ETR gene expression, its protein abundance and ethylene production rate in winter (blue) and summer fruit (red) during ripening and post-harvest storage.

**Supplementary Fig. 8.** Correlation between CTR1, CTR2, EIN2 gene expression, their protein abundance and ethylene production rate in winter (blue) and summer fruit (red) during ripening and post-harvest storage.
