# Supplementary material for: Targeted system approach to ethylene biosynthesis and signaling of a heat tolerant tomato cultivar; the impact of growing season on fruit ripening
Source: Front Plant Sci. 2023 Jun 30;14:1195020. doi: 10.3389/fpls.2023.1195020 (PMC10348052; doi:10.3389/fpls.2023.1195020)
Supplement: Supplementary file 2 [file DataSheet_2.docx]

**Supplementary Table 1.** Description of maturity stage

| **Maturity Stage** | **Description** |
| --- | --- |
| Immature green (IMG) | Almost fully expanded, the surface of the tomato is completely green in color, stigma attached |
| Mature green (MG) | Fully expanded, the surface of the tomato is completely green in color, stigma detached |
| Breaker (BR) | Not more than 10% of the surface from the center bottom shows tannish- yellow, orange or red |
| Turning (TRN) | From 10 to 30 % of the surface shows tannish-yellow or pink or red. |
| Orange (ORG) | From 30 to 60 % of the surface, in the aggregate, shows red-orange or red |
| Light red (LR) | From 60 to 90% of the surface, in the aggregate, shows red-orange or light red |
| Red ripe (RR) | More than 90% of the surface, in the aggregate, shows red |

**Supplementary Table 2.** List of primer sequences for RT-qPCR

| **Gene name** | **Gene ID** | **Sequences** | | **Amplicon** |
| --- | --- | --- | --- | --- |
|  |  | **Forward** | **Reverse** | **(bp)** |
| ACO1 | 544052 | ACAAACAGACGGGACACGAA | CCTCTGCCTCTTTTTCAACC | 101 |
| ACO2 | 101251255 | GTCATTAGCATCCTTCTACAATCCA | TGTTATGTTCCTCTGCCTCTTTATC | 89 |
| ACO3 | 544285 | TGATTACCAACGGGAAGTACAAG | CAATTAGAGATGGTGCTGGATAGA | 132 |
| ACO4 | 543506 | TGATTGAGAAGACAGAAGAGGACA | GGAATTTGAGACCTGCATACAAC | 90 |
| ACO5 | 543800 | ACTACGAAGCCAACATGAAGAAG | GACGATGCCAAACAAAGAATG | 117 |
| ACO6 | 100125909 | ACCAGAATTGCTGGAGAAGG | CCTTGGCTCTTTAGCTTGGA | 115 |
| ACO7 | 101266529 | TGATGCTGGTGGTGTCATTT | CACGATGGCGTTAGGGATAG | 109 |
| ACS1A | 544028 | ACACAACATTTGCTTGCATC | AAACATCCCCTGCCTTTCTC | 99 |
| ACS1B | 101246650 | TGTTCACAAAAGGACTAGCACAA | AAGCAGTCTCCTTAAATCCATCC | 89 |
| ACS2 | 606304 | TCTAAGAGAAAGCGCGATGAG | AAAAGCCCCGCATTATTTTT | 102 |
| ACS3 | 778292 | GCTGGTTCCGTGTATGTTTTG | CTGGTGATTAGTTGCGTTGG | 110 |
| ACS4 | 778356 | AAACGTGTAGTAATGGCTGGTG | AAATCCTGGGTAATAGGGTGTG | 111 |
| ACS5 | 606300 | ATGTATCCCCTGGTTCCTCCT | CGCGAGGTCTAGTGTTTCTTTT | 92 |
| ACS6 | 606302 | CAATACTGTAGAACAAGGAGCAAAC | GACCATAGGAGAAGACATAGGTGAA | 102 |
| ACS7 | 543621 | CGTGTCTGAAAATCAAAAGAGG | TGTCTCATATCCACCCAACAAA | 123 |
| ACS8 | 606301 | TATTTGCCAAGTGGGGTTTT | CACCATGGACAAGGGAATGT | 111 |
| ETR1 | 606298 | TGAATCTAAGCTCCCCTTTGTG | TCTCATCCATCACCAAAACCT | 89 |
| ETR2 | 606299 | ACCAGGAACCAGCAACTAAAAA | CGACACCTTCACTTTCAATCC | 111 |
| ETR3 | 544279 | ATTCTCCGTCGTCATTTTTCAC | GAGTTTCCTTCCTTCCTTCCTT | 119 |
| ETR4 | 543588 | CGTGAATAGAGCGGTAACAAGTAAG | AGGGCTAAGAACACCAATACA | 98 |
| ETR5 | 543589 | TGGGCCCTTCACTAACAACC | ACTACGGCTGCGGAACTTAC | 107 |
| ETR6 | 543834 | CCCGTTGTGTCCTCGTTACA | CCGATGATCAAAGGCCAGGT | 119 |
| ETR7 | 101249081 | ATACCGATGGCCATGCACAA | TCGCTTGCTCCAAAGGATGT | 105 |
| CTR1 | 544127 | GCATATCCCCTAGTTGCATCAC | CATGGAAACCAGTTCCTCTTCT | 113 |
| CTR2 | 543725 | TGCAAGCTCAGTCAATAGGAAC | ACCAACATCATCAAACACAGGA | 110 |
| CTR3 | 543907 | ATTTGTTCGGTGGCTCGTCT | CAGCACAGGTAGCTTCGGAA | 108 |
| CTR4 | 543908 | TGCTGGACAAACGGACAGAA | TTGAAGGCAAAGGTAGCCGT | 95 |
| EIN2 | 543928 | GTCCGGGGTCATACAAAAGTCT | GAGCTTCTGTTAACTGCCTCCT | 117 |
| ACT | AB199316 | ACAGGTGTTATGGTCGGAATG | GCTCAATGGGATATTTCAAGGT | 97 |
| EF1 | BT012693 | TTGGTCATGTTGACTCTGGAAA | CCTTCTCGAACCTCTCAATAACAC | 116 |
| GAPDH | X14449 | CTAAAGGTCAAGGATGAGAAGA | CAACAACGAAGTCAGCACCA | 96 |
| RPL2 | X64562 | CCGATTCCGTACTCTCGATTT | GCTCCTTCTGATGCTTGTACCT | 143 |

**Supplementary Table 3.** List of unique peptides for protein identification

| **Peptide sequences** | **Protein corresponding to** | **Entry number** | **Types of modification** |
| --- | --- | --- | --- |
| ANTMEMIK | ACO1 | P05116 |  |
| ANT**M**EMIK | ACO1 | P05116 | Oxidation (1st Met) |
| ANTME**M**IK | ACO1 | P05116 | Oxidation (2nd Met) |
| ANT**M**E**M**IK | ACO1 | P05116 | Oxidation (both Met) |
| MSLASFYNPGSDAVIYPAK | ACO1 | P05116 |  |
| **M**SLASFYNPGSDAVIYPAK | ACO1 | P05116 | Oxidation (Met) |
| EAEESTQVYPK | ACO1 | P05116 |  |
| GLEGVEVEVTDMDWESTFFLR | ACO2 | P07920 |  |
| GLEGVEVEVTD**M**DWESTFFLR | ACO2 | P07920 | Oxidation (Met) |
| NTFYGSK | ACO2 | P07920 |  |
| GLEAVQTEIDDLDWESTFFLK | ACO4 | Q9ZWP2 |  |
| LAENLLDLL**C**ENLGLEK | ACO4 | Q9ZWP2 | Carbamidomethyl (Cys) |
| VIAQQDGTR | ACO4 | Q9ZWP2 |  |
| AVETTVNLGPIETV | ACO4 | Q9ZWP2 |  |
| AVDDYIDQLIK | ACO5 | Q6A1K7 |  |
| EAFSGSK | ACO5 | Q6A1K7 |  |
| GPSVGTK | ACO5 | Q6A1K7 |  |
| LSIATFYNPAGEAIISPASK | ACO5 | Q6A1K7 |  |
| FQDYLNLYSK | ACO5 | Q6A1K7 |  |
| LLYP**C**HLR | ACO5 | Q6A1K7 | Carbamidomethyl (Cys) |
| PASNIYEIQGLSK | ACO5 | Q6A1K7 |  |
| GLEGVQTEIDDLDWESTFFLK | ACO6 | A4ZYQ6 |  |
| LAEQLLDLL**C**ENLGLEQGYLK | ACO6 | A4ZYQ6 | Carbamidomethyl (Cys) |
| VIAQPDGNR | ACO6 | A4ZYQ6 |  |
| AVETAVNLGPIATV | ACO6 | A4ZYQ6 |  |
| AQTLAQISK | ACO7 | A0A3Q7GUK8 |  |
| VGEE**C**FK | ACO7 | A0A3Q7GUK8 | Carbamidomethyl (Cys) |
| VVNLLNEFVESNK | ACO7 | A0A3Q7GUK8 |  |
| AFNNNNNNDAFFGTK | ACO7 | A0A3Q7GUK8 |  |
| SIASFYNPSLK | ACO7 | A0A3Q7GUK8 |  |
| ATISPAPELLILEEK | ACO7 | A0A3Q7GUK8 |  |
| FQAVAAI | ACO7 | A0A3Q7GUK8 |  |
| VGIVYSYNDTVVNIAR | ACS1A | Q9S854 |  |
| GLAEVGISTLK | ACS1A | Q9S854 |  |
| EATFDSELELWR | ACS1A | Q9S854 |  |
| ISYFVLQPK | ACS1A | Q9S854 |  |
| VGIVYSYNDTVVNISR | ACS1B | Q9S853 |  |
| GLAQVGISTLK | ACS1B | Q9S853 |  |
| EATFDGELELWR | ACS1B | Q9S853 |  |
| NFVLQTK | ACS1B | Q9S853 |  |
| TNSILSK | ACS2 | P18485 |  |
| GSI**C**SEGIK | ACS2 | P18485 | Carbamidomethyl (Cys) |
| EAYENAQK | ACS2 | P18485 |  |
| GLILTNPSNPLGTTLDK | ACS2 | P18485 |  |
| VGIIYSFNDDVVN**C**AR | ACS2 | P18485 | Carbamidomethyl (Cys) |
| FVDNFLR | ACS2 | P18485 |  |
| VIINDVK | ACS2 | P18485 |  |
| ITESALEEAYLDAK | ACS3 | Q42881 |  |
| GVLVTNPSNPLGTTLNR | ACS3 | Q42881 |  |
| NELELLLTFIDEK | ACS3 | Q42881 |  |
| SSVVLSK | ACS4 | P29535 |  |
| ASI**C**TNDGIESFR | ACS4 | P29535 | Carbamidomethyl (Cys) |
| SGVQLLPIS**C**K | ACS4 | P29535 | Carbamidomethyl (Cys) |
| ITIEAIEEAYEK | ACS4 | P29535 |  |
| GQQANVK | ACS4 | P29535 |  |
| GLILTNP**C**NPLGTILDR | ACS4 | P29535 | Carbamidomethyl (Cys) |
| FVSIAEIINEDN**C**INK | ACS4 | P29535 | Carbamidomethyl (Cys) |
| VGIVYSFNDDVVN**C**AR | ACS4 | P29535 | Carbamidomethyl (Cys) |
| GLEEIGIK | ACS4 | P29535 |  |
| LIINEVK | ACS4 | P29535 |  |
| NQDVIQFR | ACS5 | Q94GA4 |  |
| TGAEIVPIQ**C**YSSNNFR | ACS5 | Q94GA4 | Carbamidomethyl (Cys) |
| ITESALEEAYEQAQK | ACS5 | Q94GA4 |  |
| FVSIIEALIDR | ACS5 | Q94GA4 |  |
| INNFVNFDR | ACS5 | Q94GA4 |  |
| FVTGAGSR | ACS5 | Q94GA4 |  |
| TIANWVVK | ACS5 | Q94GA4 |  |
| TGVQLFPVV**C**ESSNNFK | ACS6 | Q9SAZ4 | Carbamidomethyl (Cys) |
| EALEEAYSK | ACS6 | Q9SAZ4 |  |
| AQESNIK | ACS6 | Q9SAZ4 |  |
| GLLINNPSNPLGTILDK | ACS6 | Q9SAZ4 |  |
| VGIIYSYNDAVVNIAR | ACS6 | Q9SAZ4 |  |
| GLGQVGITTLK | ACS6 | Q9SAZ4 |  |
| FLENSTFDDELK | ACS6 | Q9SAZ4 |  |
| GVEVATK | ACS6 | Q9SAZ4 |  |
| LEISLSFR | ACS6 | Q9SAZ4 |  |
| NPYDEIR | ACS7 | O81143 |  |
| VSFDSNK | ACS7 | O81143 |  |
| ITESALEEAYTEAER | ACS7 | O81143 |  |
| GVLVTNPSNPLGTTLTK | ACS7 | O81143 |  |
| ELQLLLTFVSTK | ACS7 | O81143 |  |
| VGAIYSNDDR | ACS7 | O81143 |  |
| NYVSENQK | ACS7 | O81143 |  |
| VTFNPNK | ACS8 | Q94GA3 |  |
| ITQSALEESYK | ACS8 | Q94GA3 |  |
| GILVTNPSNPLGTTLTR | ACS8 | Q94GA3 |  |
| NELELLVSFVAEK | ACS8 | Q94GA3 |  |
| IGAIYSNDAVIVSAATK | ACS8 | Q94GA3 |  |
| TNTFEAEIELWK | ACS8 | Q94GA3 |  |
| NAFYGSK | ACO1 & ACO3 | P05116 & P24157 |  |
| VIAQTDGTR | ACO1 and ACO3 | P05116 & P24157 |  |

**Supplementary Table 4.** Heavy labeled peptides with known concentration

| **Peptide** | **Protein** | **Amount used for quantification (fmol/µg peptide)** |
| --- | --- | --- |
| MSLASFYNPGSDAVIYPAK* | ACO1 | 500 |
| **M**SLASFYNPGSDAVIYPAK* | ACO1 | 400 |
| NTFYGSK* | ACO2 | 400 |
| LSIATFYNPAGEAIISPASK* | ACO5 | 500 |
| FQDYLNLYSK* | ACO5 | 10 |
| AVDDYIDQLIK* | ACO5 | 200 |
| AVETAVNL*GPIATV | ACO6 | 400 |
| ISPNSPVAR* | ETR1 | 10 |
| EGNVSISAFVAK* | ETR1 | 50 |
| YIPGEVVAVR* | ETR2 | 5 |
| YIPPEVVAVR* | ETR3 | 10 |
| VPLLHLSNFTNDWAELSTR* | ETR3 | 100 |
| LIQTLLNVAGNAVK* | ETR3 | 400 |
| DSSFNSAYNLPIPR* | ETR4 | 15 |
| SDPDVIQVK* | ETR4 | 15 |
| VLPESVSR* | ETR4 | 10 |
| SLSINDPDVLEITK* | ETR5 | 50 |
| FWLNQEVEIVR* | ETR6 | 25 |
| GVEVLLADYDDSNR* | ETR6 | 100 |
| SLPIDDPDVLEITK* | ETR7 | 15 |
| GLQVLLADDDDVNR* | ETR7 | 100 |
| IPSIESLR* | CTR1 | 15 |
| LNPPQVIAAVGFNR* | CTR1 | 15 |
| YAPNEVPR* | CTR2 | 10 |
| LVIPAYVDQLNSR* | CTR2 | 10 |
| ASASAASAETLSHR* | CTR3 | 5 |
| GVSENAQSFISDGPGSYK* | EIN2 | 50 |
| VESSAYIPSGSAR* | EIN2 | 5 |

* heavy labelled animo acid
